# Development and validation of a rapid and robust method to determine visceral adipose tissue volume using computed tomography images

**DOI:** 10.1371/journal.pone.0183515

**Published:** 2017-08-31

**Authors:** Aaroh M. Parikh, Adriana M. Coletta, Z. Henry Yu, Gaiane M. Rauch, Joey P. Cheung, Laurence E. Court, Ann H. Klopp

**Affiliations:** 1 Department of Physics and Astronomy, Rice University, Houston, Texas, United States of America; 2 Department of Behavioral Science, The University of Texas M.D. Anderson Cancer Center, Houston, Texas, United States of America; 3 Department of Radiation Physics, The University of Texas M.D. Anderson Cancer Center, Houston, Texas, United States of America; 4 Department of Radiation Oncology, Christiana Care Health System, Newark, Delaware, United States of America; 5 Department of Diagnostic Radiology, The University of Texas M.D. Anderson Cancer Center, Houston, Texas, United States of America; 6 Department of Radiation Oncology, The University of Texas M.D. Anderson Cancer Center, Houston, Texas, United States of America; INIA, SPAIN

## Abstract

**Background:**

Visceral adiposity is a risk factor for many chronic diseases. Existing methods to quantify visceral adipose tissue volume using computed tomographic (CT) images often use a single slice, are manual, and are time consuming, making them impractical for large population studies. We developed and validated a method to accurately, rapidly, and robustly measure visceral adipose tissue volume using CT images.

**Methods:**

In-house software, Medical Executable for the Efficient and Robust Quantification of Adipose Tissue (MEERQAT), was developed to calculate visceral adipose tissue volume using a series of CT images within a manually identified region of interest. To distinguish visceral and subcutaneous adipose tissue, ellipses are drawn through the rectus abdominis and transverse abdominis using manual and automatic processes. Visceral and subcutaneous adipose tissue volumes are calculated by counting the numbers of voxels corresponding to adipose tissue in the region of interest. MEERQAT’s ellipse interpolation method was validated by comparing visceral adipose volume from 10 patients’ CT scans with corresponding results from manually delineated scans. Accuracy of visceral adipose quantification was tested using a phantom consisting of animal fat and tissues. Robustness of the method was tested by determining intra-observer and inter-observer coefficients of variation (CV).

**Results:**

The mean difference in visceral adipose tissue volume between manual and elliptical delineation methods was -0.54 ± 4.81%. In the phantom, our measurement differed from the known adipose volume by ≤ 7.5% for all scanning parameters. Mean inter-observer CV for visceral adipose tissue volume was 0.085, and mean intra-observer CV for visceral adipose tissue volume was 0.059.

**Conclusions:**

We have developed and validated a robust method of accurately and quickly determining visceral adipose tissue volume in any defined region of interest using CT imaging.

## Introduction

Excess visceral adipose tissue increases the risk of diabetes, cardiovascular disease, and several types of cancer [1–4]. A method to reliably and rapidly measure visceral adipose tissue volume may provide important prognostic information and be of use within both clinical and research settings. Historically, visceral adiposity is estimated using anthropometric methods that measure total abdominal adiposity, such as waist circumference and sagittal abdominal diameter [5]. Anthropometric methods are unable to directly measure visceral adipose tissue (VAT) volume, nor can they distinguish between VAT and subcutaneous adipose tissue (SAT) volume [6]. For VAT and SAT to be quantified independently, volumetric imaging methods such as magnetic resonance imaging (MRI) and computed tomography (CT) must be used; however, current MRI- and CT-based methods are not without limitations.

While a benefit of MRI includes the use of nonionizing radiation, a limitation is that it requires long acquisition times, making it prone to motion artifacts. Further, MRI is a very expensive procedure and is not accessible to a large segment of the population. Regarding CT, most methods examine a single axial CT slice at the umbilical level to *estimate* VAT and SAT volumes using two-dimensional measurements [7, 8]. A limitation of using single-slice measurements to estimate volume is that they can be greatly affected by bowel contents, especially when large amounts of gas are present in the small and large intestines [9]. Researchers have shown that planimetric estimates yield significantly different SAT volume to VAT volume ratios than volumetric measurements [10, 11]. Furthermore, separation of VAT, SAT, and other organs using CT analysis is typically a manual process requiring many man-hours [10, 12]. As a result, existing approaches to quantifying VAT with CT do not use volumetric methods when large numbers of patients are analyzed.

Although CT scans expose patients to ionizing radiation, as compared to MRI, CT scans may be performed in less time with fewer image artifacts, are less expensive, and are more widely used. Therefore, the use of CT to measure VAT volume may be more promising than the use of MRI. Thus, the purpose of the present investigation was to develop a volumetric method and software program to accurately and rapidly calculate VAT and SAT volume in any defined region of interest using an abdominal CT scan. Accordingly, we improved upon current CT-based methods of VAT and SAT volume calculation by minimizing manual interaction, automating and streamlining the process, and accurately calculating adipose volumes. Furthermore, we used a tissue phantom in order to validate the Hounsfield Unit (HU) range for identifying adipose tissue (-190 to -30 HU).

## Materials and methods

### Patient cohort

A retrospective review of abdominal CT images from 10 patients was conducted. All patients were women between the ages of 45 and 85 years (mean BMI 23.8 ± 7.7 kg/m^2^, BMI range: 14.6–36.9 kg/m^2^) with stage II-IV ovarian cancer treated between 2000 and 2010. All CT images were acquired using a GE Lightspeed CT scanner (General Electronics Healthcare, Milwaukee, WI), with a tube voltage of 120 kilovoltage peak (kVp) and a field of view (FOV) of 50 cm. Tube current varied between 265 milliamperage (mA) and 300 mA, and slice thickness was either 2.5 mm or 5 mm. Patients were selected on the basis of their body mass index (BMI). Five patients with BMI < 25 kg/m^2^ were considered “lean” and the other five patients with BMI ≥ 30 kg/m^2^ were considered “obese.” The robustness of our method was tested by selecting patients at both extremes. The protocol for the present investigation was approved by the University of Texas M.D. Anderson Cancer Center Institutional Review Board.

### In-house adipose volume calculation program

**Fig 1:**
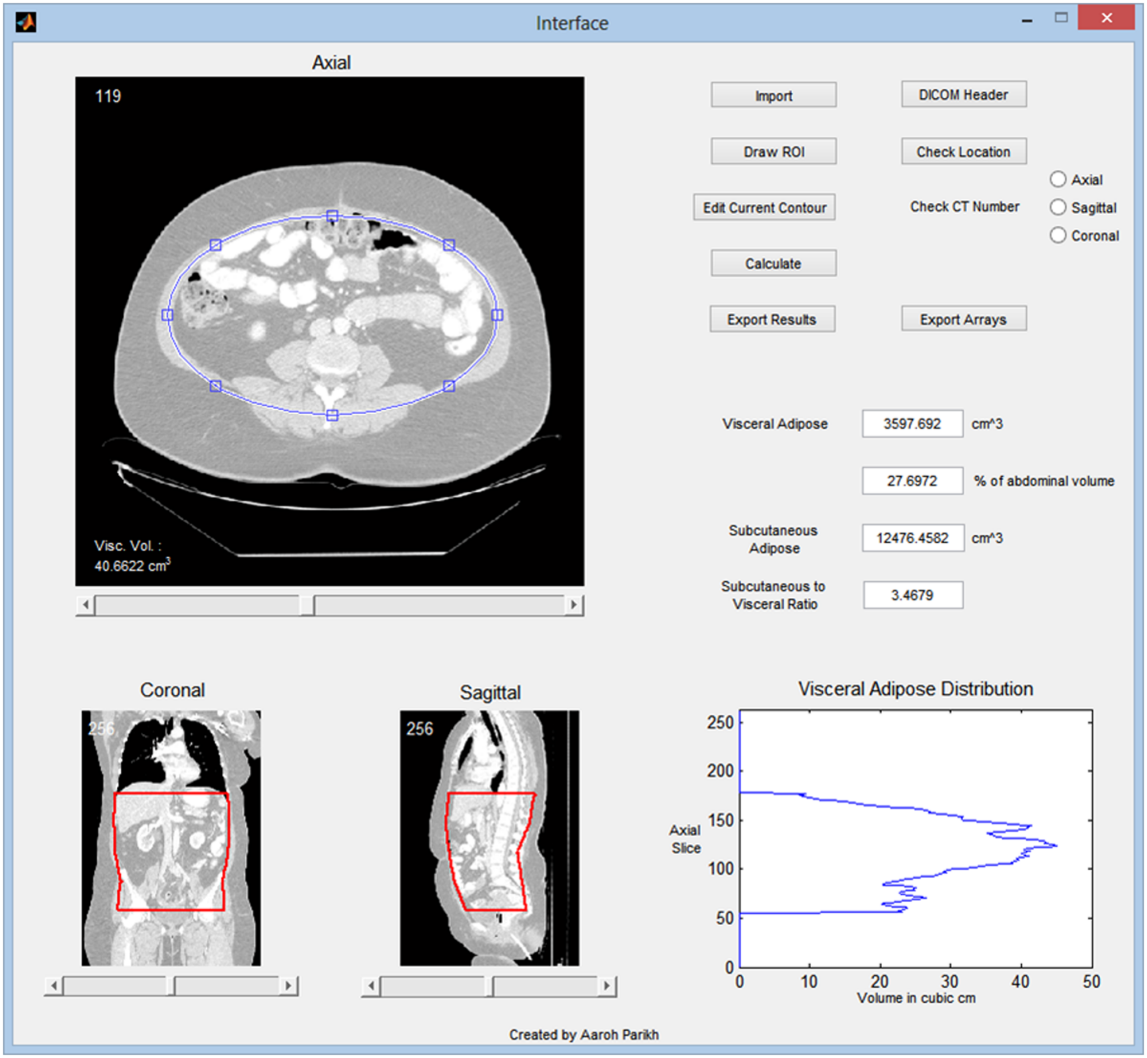
Graphic interface of MEERQAT. Axial, coronal, and sagittal views of the computed tomography (CT) image series are displayed on the left. Ellipses are drawn on axial slices (blue line), and interpolated ellipses are visible on the coronal and sagittal displays. Visceral and subcutaneous adipose tissue volumes are shown on the right, along with a plot of visceral adipose tissue volume per slice.

MATLAB™ (Mathworks, Natick, MA) was used to create an in-house program, Medical Executable for the Efficient and Robust Quantification of Adipose Tissue (MEERQAT), to calculate adipose volume. For each patient’s abdominal CT scan, the CT images were imported into MEERQAT as a series of DICOM images. The images were then reconstructed into a three-dimensional volume and displayed in the axial, coronal, and sagittal viewing planes (Fig 1). To limit the analysis in MEERQAT to a specific area, the user can manually define the upper and lower boundaries to a region of interest (ROI). Because this study is focused on abdominal adipose tissue, the upper and lower boundaries of the ROI were defined from the bottom of the diaphragm to the superior aspect of the femoral heads.

**Fig 2:**
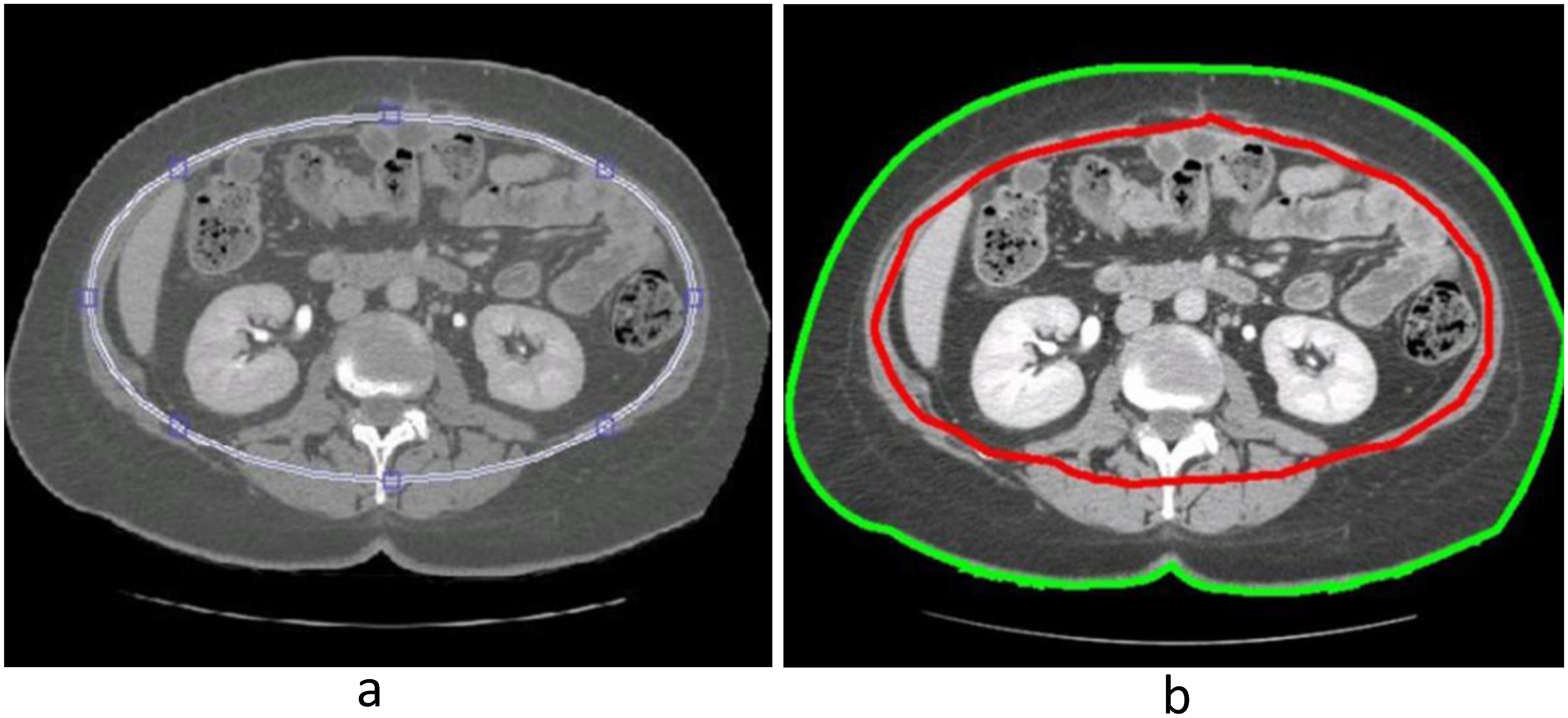
Contouring methods for the separation of subcutaneous and visceral adipose tissue. (a) In MEERQAT (our program), elliptical contours, shown in blue, were used to divide VAT and SAT regions within each slice. (b) In Pinnacle^3^ (typical program used to delineate VAT and SAT regions), two manual contours, shown in red and green, were drawn on each slice to separate the VAT region from the SAT region.

Next, the user must delineate a structure to separate VAT and SAT. To accomplish this task, elliptical contours were shaped inside the rectus abdominis and transverse abdominis extending posteriorly to the vertebral body so that VAT was contained within the ellipse and SAT was outside the ellipse (Fig 2a). The ellipses were only drawn on the upper, middle, and lower quartiles, and the top and bottom slices of the ROI. During this process, the optimal location and size of the ellipse to separate VAT and SAT was determined. The program then linearly interpolated these contours for the remainder of the slices, effectively separating the three-dimensional ROI into two regions: the area enclosed by the ellipse contained the abdominal cavity, which included VAT and organs; the area outside of the ellipse contained SAT, skin, the CT couch, and air outside of the patient.

Finally, in accordance with established methods, the program calculated total adipose tissue volume by automatically counting the number of voxels between -190 and -30 Hounsfield units (HU) and multiplying this total by the volume of each voxel. The HU ranges were chosen according to the standard of practice [7, 13–16]. Because the visceral and subcutaneous regions were separated, the program was able to calculate VAT volume and SAT volume independently.

### Elliptical interpolation versus manual delineation

To evaluate our method of separating VAT and SAT using ellipses, we calculated VAT volume and SAT volume for the 10 patients using ellipse interpolation (our method) and manual delineation. Manual delineation was performed by drawing two contours on every slice using contouring tools available in Pinnacle^3^ Treatment Planning Software for Radiation Therapy (Philips Healthcare, Andover, MA). The shape of these contours could be arbitrary because they were not required to conform to any designated shape. The outer contour traced the skin of the patient and the inner contour traced the outer margin of the peritoneal contents (Fig 2b). The region inside the inner contour contained VAT and the region between the two contours contained SAT. Our ellipse interpolation method was performed as described in the previous section. VAT volume was then calculated for each patient using the two contouring methods and by counting voxels between -190 and -30 HU. Differences between the two methods were quantified.

### Validation of volume calculation

As stated previously, current methods quantify adipose tissue by setting a voxel threshold between -190 and -30 HU. In order to quantify the accuracy of our method, we wanted to validate the current standard by creating a phantom that closely mimicked human visceral contents. The use of a tissue phantom provided the ground truth of exactly how much adipose tissue was within. The accuracy of the fixed HU threshold was determined by comparing the known volume to the calculated adipose volume from CT images and MEERQAT. To construct a realistic phantom, the following was used: fresh pig belly fat, pig liver, pig heart, chicken drumsticks (containing muscle and bone), and water. It was especially crucial to obtain fresh animal fat because adipose tissue consistency changes irreversibly once it is cooled to refrigeration temperature, at which point it is no longer comparable to human adipose tissue. The ingredients were then mixed together in a 1.9 liter plastic container and imaged using a GE Discovery CT Scanner (General Electronics Healthcare, Milwaukee, WI).

To establish a ground truth, the volume of belly fat was measured using a water displacement method prior to mixing. To test the accuracy of our method under different CT scanning parameters, a variety of scanning protocols was used. Tube voltage, current, slice thickness, and reconstruction field of view (FOV) were altered individually for each scan. The baseline CT scan of the phantom was taken at 120 kVp and 150 mA, with 2.5-mm slice thickness and 50-cm FOV. Variations included changing the tube voltage to 80 kVp, reducing the current to 50 mA, reducing the slice thickness to 1.25 mm, and increasing the FOV to 65 cm. MEERQAT was used to calculate the total adipose volume for each scanning protocol.

### Robustness testing and speed

Three independent users performed VAT volume and SAT volume calculations using images from the 10 patients and MEERQAT to test for robustness. For three of the 10 patients, each user calculated VAT volume and SAT volume five times to test intra-observer variation. To test inter-observer variation, each user calculated VAT volume a single time on the seven remaining patients. Intra-observer and inter-observer variations were determined by calculating the coefficient of variation (CV).

To determine the average time required for a user to calculate the VAT volume of a single patient using MEERQAT, each user completed all intra-observer and inter-observer variation tests in one sitting with a stopwatch. The average time required to evaluate one patient was determined by dividing the total time by 23 (15 from intra-observer variation tests and 7 from inter-observer variation tests).

## Results

### Validation of elliptical contours

The manual delineation method required approximately 15 minutes per patient, on average, to determine VAT volume. In contrast, the ellipse interpolation method in MEERQAT, required about 2.5 minutes per patient; this time includes the entire process: importation of CT images, definition of the ROI, ellipse delineation, and calculation of VAT and SAT volumes. The mean difference (mean ± standard deviation) between VAT volume obtained from elliptical contours and VAT volume obtained from manual delineation for all patients was -0.54 ± 4.81% (Table 1). For 9 out of 10 patients, this difference was less than 5%. Separating the patients by BMI, the mean differences were -3.0 ± 4.7% for lean patients and 1.9 ± 3.9% for obese patients (S1 Data).

**Table 1:** VAT volume from elliptical interpolation & manual delineation.

Patient	Manual delineation (cm^3^)	Ellipse interpolation (cm^3^)	Absolute difference (cm^3^)	Percent difference
Lean				
1	270.8	261.5	-9.30	-3.43
2	299.9	312.8	12.90	4.30
3	1278.9	1220.2	-58.70	-4.59
4	840.7	769.3	-71.40	-8.49
5	1385.3	1345.4	-39.90	-2.88
Obese				
6	6110.7	6042.7	-68.00	-1.11
7	4285.6	4438.3	152.70	3.56
8	3470.8	3523.2	52.40	1.51
9	3649.4	3579.5	-69.90	-1.92
10	3586.0	3861.7	275.70	7.69
Mean			17.65	-0.54
Standard deviation			114.92	4.81

VAT = visceral adipose tissue.

### Validation of volume calculation using the phantom

**Fig 3:**
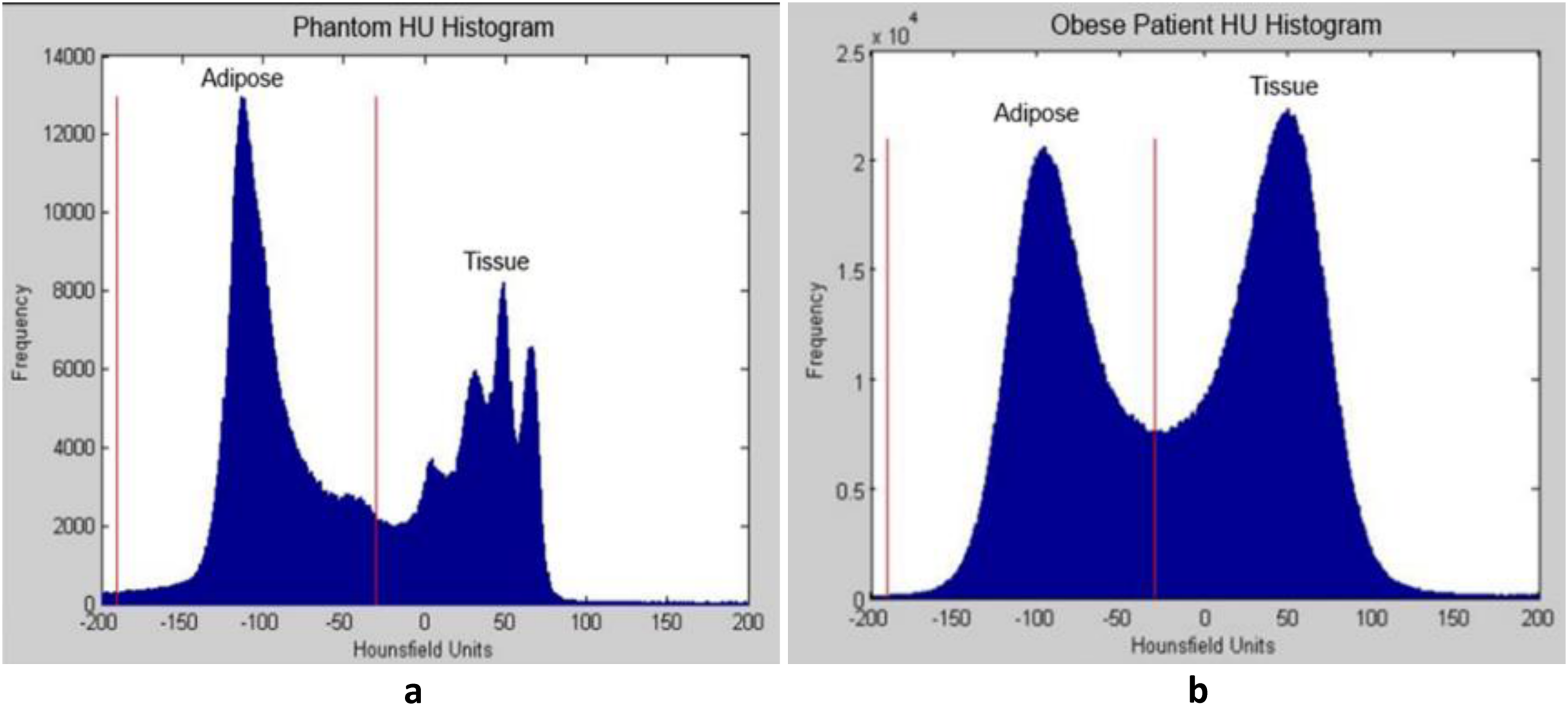
Comparison between phantom and patient Hounsfield unit (HU) distribution. (a) The HU histogram of the phantom. (b) The HU histogram of an obese patient.

The absolute volume of adipose tissue in the phantom was 1300 cm^3^. The phantom contained an HU distribution that was narrower than that of an obese human (Fig 3). Using the images obtained from the baseline CT scanning protocol of 120 kVp, 150 mA, 2.5-mm slice thickness, and 50-cm FOV, the difference in calculated adipose volume from the ground truth was 5.8%. Decreasing the tube voltage decreased accuracy, and decreasing the slice thickness or FOV increased accuracy (Table 2) (S1 Data).

**Table 2:** Phantom scanning protocols and calculated adipose volumes.

kVp	mA	Slice thickness (mm)	Field of view (cm)	Calculated adipose volume (cm^3^)	Percent difference
120	150	2.5	50	1375.80	5.83
80	150	2.5	50	1397.00	7.46
120	50	2.5	50	1376.20	5.86
120	150	1.25	50	1369.90	5.38
120	150	2.5	65	1379.20	6.09

kVp = kilovoltage peak; mA = milliamperage.

### Robustness of the ellipse interpolation method

The mean intra-observer CVs were 0.059 for VAT volume and 0.021 for SAT volume (Table 3). The mean inter-observer CVs were 0.085 for VAT volume and 0.042 for SAT volume (Table 4) (S1 Data).

**Table 3:** Intra-observer CV for VAT volume and SAT volume.

Patient	CV for VAT volume				CV for SAT volume			
	User 1	User 2	User 3	All users	User 1	User 2	User 3	All users
1	0.031	0.055	0.351		0.013	0.028	0.082	
2	0.007	0.021	0.018		0.003	0.019	0.019	
3	0.009	0.015	0.024		0.009	0.007	0.01	
Mean				0.059				0.021

CV = coefficient of variation, VAT = visceral adipose tissue, SAT = subcutaneous adipose tissue.

**Table 4:** Inter-observer CV for VAT volume and SAT volume.

Patient	VAT volume	SAT volume
1	0.177	0.050
2	0.137	0.030
3	0.108	0.012
4	0.118	0.064
5	0.036	0.031
6	0.060	0.061
7	0.113	0.052
8	0.036	0.058
9	0.026	0.037
10	0.038	0.027
Mean	0.085	0.042

CV = coefficient of variation, VAT = visceral adipose tissue, SAT = subcutaneous adipose tissue.

## Discussion and conclusion

The present investigation created and validated a method (ellipse interpolation) and software program (MEERQAT) that accurately and quickly measures VAT volume using a series of abdominal CT images. MEERQAT’s ellipse interpolation method sacrificed accuracy by less than 5% in most patients, thereby validating the use of ellipse interpolation within the MEERQAT program. With a calculation speed of 2.5 minutes per patient and an average volume difference of 0.5% from manual delineation, this method constitutes a substantial improvement over existing approaches. While the ellipse interpolation method yielded a smaller mean percent difference among obese patients compared to lean patients, this may be due to the fact that percent errors in VAT volume measurement are magnified in patients with smaller total VAT volume.

This is the first study, to our knowledge, to use a tissue phantom in order to validate the use of the -190 to -30 HU range, which is the current standard of practice. MEERQAT calculated adipose volumes within 5–8% of the ground truth depending on the scanning parameters used. We hypothesize that the error range exceeded 5% due to the extreme difficulty of creating a phantom that imitates exact human anatomy. Our phantom consisted of a mixture of heterogeneous tissues that resulted in a much narrower HU distribution than that of a human patient. As a result, the narrow HU distribution of the phantom requires a smaller HU range to determine the adipose volume. Because we used a HU range designed for patients (-190 to -30 HU), our measured volume slightly exceeded the ground truth. Additionally, scans with smaller slice thickness and smaller FOV produced more accurate results, which is expected because decreasing the slice thickness or the FOV decreases the physical volume of each voxel, thus reducing partial volume averaging artifacts. The largest observed deviation from the ground truth occurred when the tube voltage was reduced to 80 kVp. Altering the tube voltage causes the energy of the x-ray spectrum to shift; the beam becomes “softer” or “harder” with decreasing or increasing kVp respectively, and thus will attenuate differently going through the same tissue.

In comparison to previous research, there are a limited number of automated methods proposed to quantify adipose tissue. Among the methods suggested by Chung and colleagues [17], Makrogiannis and colleagues [18], and Zhao and colleagues [19], all groups used a single CT slice, making their estimations susceptible to the previously discussed shortcomings of two-dimensional approaches. To our knowledge, only two other investigations have developed volumetric CT-based methods. Similar to our program, Ohshima and colleagues [20] and Nemoto and colleagues [21] included an automated volumetric CT-based method that utilized the range of -190 to -30 HU to identify adipose tissue. In contrast to the present investigation, Ohshima and colleagues [20] did not compare their automated approach to manual delineation, thus preventing any assessment of the accuracy of their method. Furthermore, they did not assess intra- and inter-observer variations or provide the speed of their calculation method, suggesting that their approach is case-specific and may not be generalizable [20]. Meanwhile, the approach developed by Nemoto and colleagues [21] resulted in a 4.25% average error rate in VAT volume compared to manual delineation, whereas our program demonstrated an analogous error rate of approximately 0.5%. The automated approach proposed by Nemoto and colleagues [21] requires more than 4-minutes of calculation time per patient, as compared to only 2.5-minutes with our program. Lastly, their approach is prone to overestimation of VAT volume due to the inclusion of adipose tissue between layers of the abdominal musculature [21]. In MEERQAT, ellipses can be drawn on the inner surface of the abdominal musculature in order to avoid counting intramuscular fat as VAT.

Strengths of the present investigation include: use of an automated and volumetric method to quantify visceral and subcutaneous adipose tissue; development of an intuitive user-friendly software program to assess adipose tissue volume; comparison of our new approach with a manual delineation control to determine the accuracy of our method; comparison of measurements between different users to demonstrate the robustness of our method; and validation of the standard HU range corresponding to adipose tissue. Nevertheless, this investigation is not without limitations, which include a small sample size of patients within the comparison analyses and the use of only females. Future research should consider testing this method and software program among a larger sample, inclusion of men, and inclusion of overweight men and women (BMI 25–30 kg/m^2^).

In conclusion, findings from the present investigation provide an accurate, rapid, and reproducible method to measure abdominal VAT volume that provides substantial improvements over comparable approaches. MEERQAT’s accuracy (percent error of 0.5%) is a whole order of magnitude smaller than existing methods, and is accurate for female patients with a BMI range from 14.6 to 36.9kg/m^2^. A quick and accurate method of measuring VAT volume may not only provide physicians with important prognostic information about individual patients, but also allows researchers to analyze large volumes of patients to discover new relationships between VAT volume and disease.

## Supporting information

S1 Data Data set for presented work. (XLSX)
